# Pattern recognition in living cells through the lens of machine learning

**DOI:** 10.1098/rsob.240377

**Published:** 2025-07-16

**Authors:** Frank Britto Bisso, Rodrigo Aguilar, Durga Shree, Yinan Zhu, Mijael Espinoza, Benjamin Diaz, Christian Cuba Samaniego

**Affiliations:** ^1^ Ray and Stephanie Lane Computational Biology Department, Carnegie Mellon University, Pittsburgh, PA, USA; ^2^ Universidad Nacional Autonoma de Mexico Escuela Nacional de Estudios Superiores Unidad Juriquilla, Queretaro, Mexico; ^3^ Vellore Institute of Technology, Vellore, Tamil Nadu, India; ^4^ Universidad Nacional Mayor de San Marcos, Lima, Peru

**Keywords:** biocomputation, neural networks, synthetic biology, signalling pathways, pattern recognition

## Abstract

At a coarse level, pattern recognition within cells involves sensing of environmental signals by surface receptors, and activating downstream signalling pathways that ultimately drive a transcriptome response, enabling biological functions such as differentiation, migration, proliferation, apoptosis or cell-type specification. This kind of decision-making process resembles a classification task that, inspired by machine learning concepts, can be understood in terms of a decision boundary: the combination of inputs relative to the classification region defined by this boundary defines context-specific responses. In this report, we contextualize machine learning concepts within a biological framework to explore the structural and functional similarities (and differences) between artificial neural networks, signalling pathways and gene regulatory networks. We take a preliminary look at neural network architectures that may better suit biological classification tasks, explore how learning fits into this paradigm, and address the role of competitive binding in cellular computation. Altogether, we envision a new research direction at the intersection of systems and synthetic biology, advancing our understanding of the inherent computational capacities of signalling pathways and gene regulatory networks.

**Figure:**
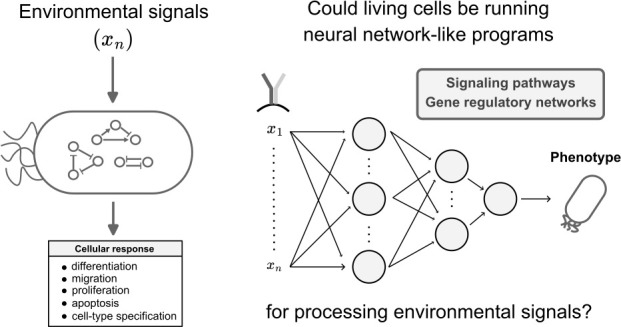

## 1. The computational principle behind cellular pattern recognition

A fundamental characteristic of cells is their ability to integrate chemical and physical signals from their environment to trigger specific responses, such as differentiation, migration, proliferation, apoptosis, or cell-type specification. These signals initiate cascades of intracellular processes regulated by signalling pathways, which serve as biological classifiers by processing input combinations to determine these context-appropriate responses. For example, during embryonic development, the TGF-β signalling pathway uses Smad transcription factors, which, upon phosphorylation and trimerization, regulate target genes that direct differentiation [1,2]. Similarly, the combinations of various cytokines processed through the JAK/STAT pathway can elicit responses ranging from inflammation and tissue repair to cell proliferation or apoptosis [3,4]. More generally, the components of signalling pathways—ligands, receptors, effector proteins and target genes—are widely expressed across different cell types, though their activation is highly context dependent [5,6]. For example, a cell expressing the same receptors may compute different biological functions in response to various ligand combinations, while the same ligands can also trigger distinct functions when interacting with different receptors [7,8]. Altogether, these interactions illustrate how signalling pathways accomplish classification tasks with high discriminatory power, enabling cells to map ligand combinations to cellular responses.

**Figure 1.**
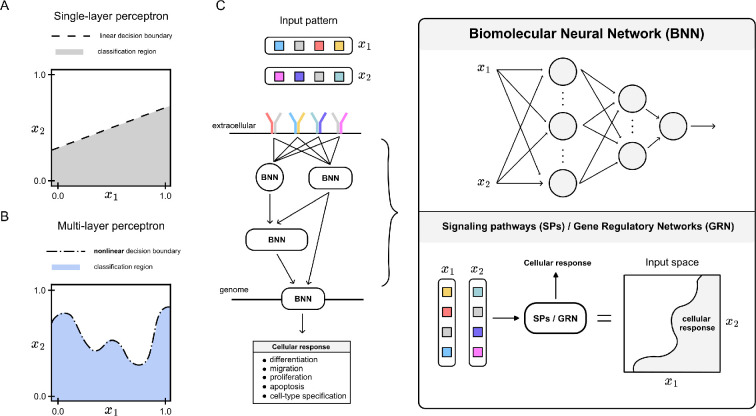
Pattern recognition in living cells. (A) Single-layer perceptrons can implement linear decision boundaries, while (B) multilayer perceptrons feature nonlinear and more complex decision boundaries. (C) For illustrative purposes, consider a simplified scenario in which environmental signals $x_{1}$ and $x_{2}$ are recognized by receptors on the cell surface, activating downstream signalling pathways that ultimately drive a transcriptome response, enabling biological functions such as differentiation, migration, proliferation, apoptosis or cell-type specification. Across various regulatory layers—including ligand–receptor interactions, downstream signal transduction and gene regulatory networks—the biological counterpart to artificial neural networks (ANNs), named biomolecular neural networks (BNNs), offers a plausible framework for understanding how information is processed to produce a cellular response. Analysing the input space for a given combination of $x_{1}$ and $x_{2}$ reveals a decision boundary that determines whether an appropriate cellular response is activated (highlighted grey area) or not.

In machine learning, classification tasks involve learning decision boundaries—surfaces that separate all possible combinations of inputs into categories—which can range from linear to complex nonlinear shapes, depending on the classification need. The simplest classifier is a perceptron, a type of neural networks that maps input values to classes through adjustable weights and creates linear decision boundaries for binary classification (figure 1A). Scaling up in complexity, multilayer perceptrons, known also as feedforward neural networks, consist of an input layer, one or more hidden layers and an output layer, allowing for complex transformations that enable nonlinear decision boundaries (figure 1B). The hidden layers and their associated activation functions introduce non-linearities that facilitate the learning of more complex mappings between inputs and outputs, transforming the input space to make non-separable data linearly separable in higher dimensions and enhancing discrimination between input combinations [9].

In this context, pattern recognition in living cells can be understood in terms of a decision boundary: the combination of inputs relative to the classification region defined by this boundary dictates the cellular response (figure 1C). BNNs have been present in synthetic biology for over a decade [10], motivated by their modularity for scaling up into more complex and autonomous decision-making systems [11]. However, the use of neuromorphic computation as a framework for modelling and understanding the pattern recognition capabilities of cellular signalling and regulatory networks at a single-cell level remains largely unexplored. This report aims to integrate fundamental machine learning concepts with a systems-level understanding of these networks to broaden the scope of biocomputation, guided by a key open question: could neural networks be the underlying computational principle behind pattern recognition in living cells?

### 1.1. Top-down perspective: signalling pathways resemble neuromorphic structures

In 1995, Dennis Bray wrote that ‘systems of interacting proteins act as neural networks trained by evolution to respond appropriately to patterns of extracellular stimuli’ [12]. This intriguing idea connecting biology and machine learning summarizes a diversity of observations on the shared hierarchical structure observed across different signalling pathways that conveys the idea of a neuromorphic structure, characterized by interconnected elements that enable computation [12,13]. Several signalling pathways, including TGF-β [14], Notch [15] and Wnt [16], are composed of multiple receptor variants which interact promiscuously with different ligands, offering versatility in the amount of cellular responses a single pathway could generate [5,17,18]. For instance, studies on the combinatorial logic of the BMP pathway reveal four different types of computation (ratiometric, additive, imbalanced and balanced) within the same set of ligands and receptor variants [19]. This ability to detect multiple signals and propagate information intracellularly resembles a neural-like structure, where individual processing units connect to one another, transmitting different information based on the combinations of signals and receptors, akin to the weighted connections between neurons. Within this analogy, the biological counterpart of the hidden layers might be the set of proteins that are activated throughout signalling cascades. For example, in the EGFR pathway, once the combination of ligands is detected, a diversity of signalling proteins are recruited by the activated receptors, resulting in phosphorylations, transmission of conformational change or close translocation to membrane-bound target molecules that ultimately target the expression of specific genes that enable the maintenance of epithelial tissue [20,21].

Furthermore, the weights in signalling pathways can be understood as the affinity between receptors and ligands, as well as the efficiency of the enzymes involved that can modulate downstream signalling proteins. In the case of BMP pathway, when a ligand binds to a protein complex formed by type I and II receptors, the type II receptor phosphorylates the type I, triggering its activation and the subsequent modification of SMAD family proteins by phosphorylation, acetylation, poly(ADP-ribosyl)ation (PARylation) and ubiquitination, which then combine with SMAD4 to form complexes with different stabilities, transcriptional activities and DNA binding affinities that translocate to the nucleus and regulate the expression of specific genes [6,22,23]. Furthermore, coreceptors such as endoglin and neuropilins play roles in modulating signal intensity, as it can associate with ligands and thus intensify or attenuate cellular functions in certain contexts [24], suggesting complementary mechanisms that enable weight tuning.

Other features shared between signalling pathways and ANNs include their design to adapt to changing stimuli, particularly during the cell differentiation, where inputs are dynamic and require highly context-specific responses [6,24,25]. Additionally, these pathways operate in a parallel, distributed manner, allowing multiple stimuli to be processed simultaneously. A clear example is the TGF-β signalling family, which senses various signals in order to trigger appropriate responses depending on the cellular context through crosstalk with other signalling pathways [6,26]. Ultimately, the interconnected network structure enables cells to ‘fine-tune’ their responses, balancing diverse signals to avoid erroneous activations and ensuring adaptability to various physiological conditions [27].

### 1.2. Bottom-up perspective: designing neural networks from scratch

The earliest *in vitro* implementation of a perceptron used toehold-mediated DNA strand displacement reactions [28,29], building on previous work with DNA hybridization reactions to implement Boolean logic gates [30]. Mimicking the activation of a neuron that turns ‘on’ when the weighted sum of its input signals exceeds a certain threshold, these DNA-based circuits reconstituted this logic by implementing multiplication, summation and comparative (thresholding) operations, all of which, when connected in a downstream fashion (from inputs to outputs), resemble a perceptron. The scale and kinetics of this implementation enabled the recognition of 4 bit patterns, which was recently scaled up to 100 bit patterns by designing a winner-takes-all circuit, where the processed inputs mutually inhibit each other, eliminating the less abundant species and restoring the dominant one’s signal [31], as well as loser-takes-all circuits, which were implemented by reversing the input signals and computing the winner-takes-all operation [32]. These systems were successful in detecting complex patterns, such as cancer genetic profiles [33,34] and handwritten characters [35], but were limited by the inability to implement negative weights due to the nature of chemical concentrations (always positive). Complementing these DNA-based implementations, recent work in de novo protein design has yielded one of the first *in vivo* neural networks [36]. This system uses proteases (enzymes that degrade other proteins) and degrons (small peptide tags that target proteins for degradation) to adjust network weights and enable tunable decision boundaries. Named ‘perceptein’, this circuit was successfully tested for controlling cell death based on an input pattern, though, like its DNA-based counterparts, it could not generate negative weights.

**Figure 2.**
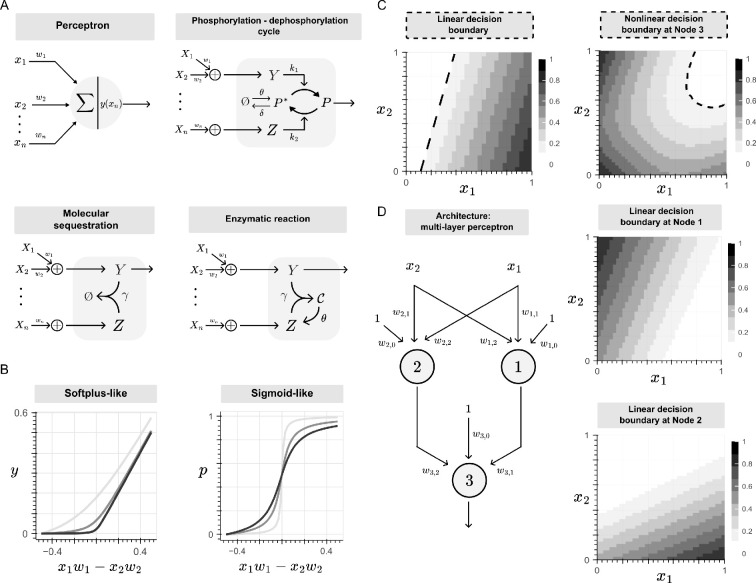
Building multilayer perceptrons using chemical reactions. Biomolecular perceptrons can be implemented through a variety of chemical reactions that are ubiquitous to signalling pahtways and gene regulatory networks: phosphorylation/dephosphorylation cycles, where a kinase *Y* adds a phosphate to the $P^{*}$ protein, and converts it into P at a rate $k_{1}$, whereas a phosphatase Z removes the phosphate of P and converts it into $P^{*}$ at a rate $k_{2}$; molecular sequestration, where a molecule Z binds to a molecule Y to form an inactive complex at a rate defined by γ; and enzymatic reactions, where a substrate Y binds to an enzyme Z to form a complex C, at a rate defined by γ, that catalyses Y’s degradation, after which Z is able to dissociate from the complex into its free form. (B) The Sotfplus-like and sigmoid-like activation functions arise when mapping the perceptron's outputs to its respective inputs, and whose shape depends on the rate constants of each reaction. (C) The single-layer perceptron exhibits a tunable decision boundary. By cascading this unit into layers, we obtain more complex, nonlinear decision boundaries. (D) Architecture of a multilayer perceptron with a hidden layer made of Node 1 (linear decision boundary on top) and Node 2 (linear decision boundary at the bottom).

Inspired by these designs, the molecular sequestration reaction (figure 2A) was introduced back in 2019 to enable ‘signed’ weights, as it approximates a subtraction operation at fast sequestration rates, resulting in both positive and negative weights [37]. This reaction was incorporated within the mathematical framework of machine learning, demonstrating a Softplus-like activation function (figure 2B) and enabling the network to function as a linear classifier (figure 2C, left) with a tunable decision boundary and classification region. Furthermore, by cascading individual perceptrons into layers, the network generates nonlinear decision boundaries (figure 2C, right). In this configuration, each perceptron computes a linear decision boundary, and the output layer combines these to create the overall nonlinear decision boundary (figure 2D).

The design of these BBNs was further extended by incorporating additional biological processes, such as the phosphorylation/dephosphorylation cycle and enzymatic reactions (figure 2A). These processes similarly exhibit activation functions (sigmoid-like and Softplus-like, respectively, as shown in figure 2B), and enable linear classifications with tunable decision boundaries and classification regions [38,39]. Moreover, the chemical species involved in these networks are ubiquitous across various regulatory layers in biological systems. For example, transcription factors such as basic leucine zippers, basic helix-loop-helix and sigma factors are commonly sequestered into inactive complexes by negative inhibitors or antagonists [40]. Gene expression is also regulated by antisense RNA strands that can degrade mRNA molecules [41]. Likewise, enzymatic reactions and post-translational modifications play key roles in regulatory networks, including gene silencing through microRNAs [42] and protein modifications such as phosphorylation, ubiquitination and cleavage [43].

Further theoretical work on the promiscuity feature of protein dimerization reactions—observed as the combinatorial binding between ligands and receptors, transcription factors and co-factors, and transmembrane adhesion proteins—has expanded the repertoire of chemical reactions with computational capabilities. These networks compute a diverse range of input–output functions by competitively forming dimers, with different functional outputs achieved solely by tuning monomer expression levels [44]. Overall, a key feature of these BNNs is the ease of scaling the complexity of the decision boundary by cascading single processing units in layers, resulting in a multilayer perceptron architecture (figures 1B and 2D). Since previous experimental implementations were constrained to pattern recognition between classes that are only linearly separable, recent efforts were focused on building a synthetic multilayer architecture by increasing the width of the hidden layer, through the use of the polymerase–exonuclease–nickase (PEN) strategy [45] and endoribonucleases [46].

### 1.3. Biomolecular neural networks: the convergence between the two perspectives

From a systems-level perspective, signalling pathways share two key features with ANN: a neuromorphic structure, exhibiting connections hierarchically ordered in layers, and the presence of a decision boundary that defines the logic behind cell decision making. Synthetic biology, using a ’build-to-learn’ approach, has mimicked perceptrons through chemical reactions that are common in cells, including molecular sequestration, post-translational modifications and enzymatic reactions. These reactions generate a tunable decision boundary whose complexity can scale by cascading them into layers, resembling a multilayer perceptron. Together, both top-down and bottom-up approaches converge on the idea that signalling pathways and gene regulatory networks possess computational capabilities, with neural networks offering a robust framework for understanding how cells recognize and respond to patterns (figure 1C). By mapping environmental signals to phenotypes, as we do with input/output mapping in engineering, the neural network model provides a framework to study cell decision making.

## 2. A first look into the similarities and differences between ANNs and BNNs

### 2.1. Analogies between neural network architectures and biomolecular neural networks

Given that the parallels between the multilayer perceptron architecture from machine learning and the computational capabilities of signalling pathways and gene regulatory networks were driven by functional similarities, an intuitive starting point for extending this analogy is to identify which type of neural network is best suited to represent the functionalities of a certain classification task in biology. Fully connected deep neural networks were the first architecture proposed to explain how combinations of transcription factors and co-factors are able to drive a specific gene expression profile, inspired by the neuromorphic characteristics of this type of network [47]. Nevertheless, research on dynamic signalling driving cell decision making [7,27] and the presence of feedback across multiple layers of genetic regulation [48] suggests that recurrent neural networks (RNNs) fit better as a model. These feedback connections enable the network to maintain an internal memory that captures information from previous inputs and updates a hidden state, which in turn influences how new inputs are processed [49]. This feature enables RNNs to excel at processing temporal patterns, where the order in which inputs are fed into the network is as important as their values. For example, recent findings on cell migration have shown that individual cells use the EGFR sensor network, constituted by negative feedback interactions [50,51], as a working memory to navigate changing chemoattractant fields, by transiently retaining information about previous signal locations through a ‘ghost [hidden] state’: a polarized state that enables persistent directional migration without compromising adaptability to new signals [52,53].

However, this transient information is likely to be lost over time, particularly in this type of system where protein modifications occur in short periods of time. As a strategy to mitigate information loss, other systems like bacteria use epigenetic switches, which allow them to retain information about environmental conditions [54,55]. This process resembles the long–short term memory (LSTM) mechanism, where information is managed through cell states, allowing the LSTM to remember or forget details over time [56]. In this case, the epigenetic modifications act as a mechanism for ‘remembering’, preserving expression patterns through generations. Conversely, the removal of chromatin marks can be linked to the ‘forgetting’ phase [57]. The retention and discarding of information is performed through a ‘gated mechanism’, which regulates positive and negative feedback. Positive feedback reinforces the cell state by retaining past information and negative feedback discards information that is no longer needed. The gating mechanism works as a complete regulatory system that balances the positive and negative feedback, enabling these networks to handle long-term dependencies.

For some specific tasks where spatial information and order are important, convolutional neural networks (CNNs) are the most suited models. These networks capture spatial relationships in the inputs by detecting and analysing local features that reflect how data are organized in space. Hidden layers—called convolutional layers—in CNNs detect local features (e.g. edges or texture of an image) by applying small filters uniformly across the entire input pattern. Then, a pooling layer aggregates these local features into a compact summary that preserves the most essential spatial details while discarding less relevant information. This summarized information is subsequently used by other layers to perform classification that considers spatial features from the input pattern [49]. A representative biological example can be observed in the formation of receptor clusters in cell membranes, which play a significant role in amplifying signals and generating specific responses based on the structure and position of the cluster [58–60]. Cells must detect and process variations in the concentration of chemotactic molecules across the membrane. However, the position of the receptors can be dynamically modulated in response to changes in ligand concentrations on the cell surface, leading to regions where the signal is stronger, thereby promoting movement in that direction [61,62]. In this way, it appears that CNNs and these biological systems operate with a similar principle: both extract spatial information from the inputs and selectively aggregate them to enable a downstream response.

Furthermore, transformers are models designed to focus on different parts of the input based on their relevance. They use a mechanism called ‘attention’, which assigns different levels of importance to each part of the input to capture dependencies [63]. One type, self-attention, allows the model to assign scores to each input based on its importance or information content. In biology, a recent study analysed the distributions of receptor combinations related to different pathways across cell types, and identified shared sets of combinations, called motifs [64]. These motifs include both shared and unique receptors, suggesting that certain key receptors drive a main response due to their specific ligand preferences, while other receptors can adjust responses to particular cellular needs or environments by selecting different types of ligands [8,65]. Another type, multi-head attention, focuses simultaneously on multiple aspects of the inputs. Similarly, cells process multiple signals beyond just ligands: they also integrate information from co-receptors [24], ions [66] or metabolites [67]. This integration of multiple signals enables the capture of different features of an input, thus allowing a more complete understanding of the patterns [68].

Finally, and for the particular case of cell–cell communication, graph neural networks (GNNs) are a suitable architecture, where nodes represent cells and each communication pathway acts as a connecting edge, representing computation at a population level. Information propagates through a mechanism called message passing, where nodes update their state based on the interactions with their neighbours [49]. For example, during cellular differentiation, each cell involved in Notch signalling can be represented as a node, characterized by the expression of Notch receptors and the Delta and Jagged ligands. Cell communication is given through their ligands and receptors interactions, resembling the edges of the network. Over time, and influenced by signals from neighbouring cells, each cell may activate the Notch receptor, triggering an intracellular signalling cascade that leads to the activation of specific genes and the selection of a differentiation fate [69,70]. This process mirrors the node state update, which in turn affects neighbouring cells. Ultimately, these local interactions lead to a coordinated outcome, observed as the differentiation of the tissue, similar to how GNNs aggregate node updates to produce a global representation of the graph.

### 2.2. Understanding learning

Another fundamental characteristic of ANN is learning, a process by which the network autonomously adjusts its weights and biases to improve performance. Specifically, a cost function is defined to track the error between the desired output and the current output. Learning is framed as the minimization of this cost function through a method known as gradient descent, where training samples are fed into the network, and the weights are adjusted via an algorithm called backpropagation until the cost function reaches a minimum [9]. Within this framework, promising implementations of learning algorithms based on chemical reactions have been proposed for training biological multilayer perceptrons [71–73]. However, no direct analogue to gradient descent or a cost function has yet been identified in a biological context, although bacterial chemotaxis [74,75] exhibits the closest behaviour to an optimization algorithm. Therefore, to explore potential mechanisms by which cells might adjust the stoichiometry of biomolecules within BNNs to adapt their phenotype to a new environment—and thereby learn—we can rely on a broad definition of learning [76].

**Figure 3.**
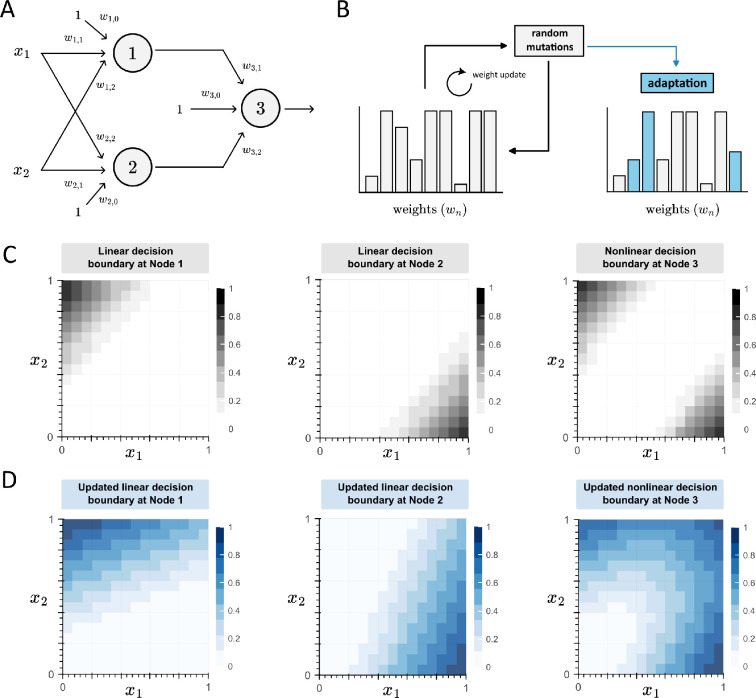
Learning is conceptualized as reshaping the decision boundary to adapt to a new environment. (A) A representative example of a multilayer perceptron processing the concentrations of molecules $x_{1}$ and $x_{2}$ to promote cell growth. (B) Evolution is framed as an iterative parameter search that introduces random mutations to the BNN, leading to adaptation. (C) Decision boundaries associated with the multilayer perceptron before perturbation of the environment. From left to right, the outputs of Node 1, Node 2 and Node 3. (D) The weights associated with Node 1 ($w_{1,n}$) and Node 2 ($w_{2,n}$) changed to that the decision boundary of Node 3 adapted to those environmental perturbations.

As a representative example, we can use the molecular sequestration reactions (figure 3A) as an example to explore a potential mechanism for learning. In this simplified scenario, a population of cells thrives in an environment where the abundance of either the $x_{1}$ or $x_{2}$ molecules promote cell growth. As illustrated in figure 3C, the decision boundary of the output layer exhibits two regions: the upper left—associated with $x_{2}$ abundance; and the bottom right—associated with $x_{1}$ abundance, both conditions support cell growth. When environmental perturbations lead to the scarcity of both molecules, survival is negatively impacted. Consequently, for adaptation, the decision boundary must be reconfigured to respond to a new combination of $x_{1}$ and $x_{2}$ molecules that support survival. As previously described (figure 1B), the nonlinear decision boundary in the output layer of a multilayer perceptron arises from the weighted sum of linear decision boundaries from each preceding layer (figure 3C, left to right). By adjusting the weights of the species involved in the sequestration reaction at a specific node—in this case, Nodes 1 and 2—we reveal a straightforward mechanism for reshaping the slope of a decision boundary, ultimately yielding a new nonlinear boundary that promotes survival (figure 3D). Notably, evolution can be then interpreted as a random search for the adequate weights for a given condition (figure 3B), where each learning iteration is a cellular generation. These weight variations will be reflected in mutations that change these production rates, either directly [77,78] or indirectly, such as varying the specificity of an enzyme to its target [79], or the number of copies of a transmembrane receptor and its binding affinity towards certain ligand [64].

### 2.3. Role of promiscuity: small networks with complex decisions

A multilayer perceptron with a single hidden layer is considered a universal function approximator, as it is able to estimate any output as a function of its inputs given a sufficient (finite) number of neurons within that layer [80]. Then, as suggested from the previous section, signalling pathways and gene regulatory networks would be working as the biological counterpart: given a sufficient number of chemical reactions, these networks would be responsible for overall cell decision making. However, although we can compare ANNs with BNNs in terms of their capacity to generate nonlinear decision boundaries, there is an inherent upper bound to the amount of functional units available for computation set by the genome size [81,82]. This raises the possibility of a mechanism allowing cells to simplify their networks without compromising the complexity of their decision boundary.

**Figure 4.**
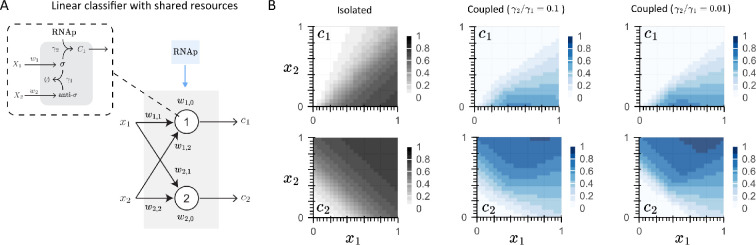
Competitive binding enables complex decision boundaries with a minimal set of reactions. (A) Schematic representation of a single-layer neural network that generates a linear classifier, where each neuron is based on the sequestration reaction between a sigma (σ) and its sigma factor (anti-σ) given at the $\gamma_{1}$ rate. The sigma factor is allowed to bind to the RNAp at a rate $\gamma_{2}$ to form a complex $c_{1,2}$ that enables transcription. (B) When isolated, each neuron presents a linear decision boundary (first column, in grey) that turns nonlinear when considering competitive binding between the sigma factor and either the anti-sigma or the RNAp, with increasing complexity when increasing the competitive binding ratios ($\gamma_{2}/\gamma_{1}$).

Among potential alternatives, competitive binding stands out as a promising candidate for that mechanism. Three recent studies have independently demonstrated how a minimal set of molecules that exhibit promiscuous interactions can generate an extensive repertoire of computations. A mathematical model for a bacterial-based single-layer perceptron built with sigma factors was developed in [83], considering a competitive dimerization reaction between a sigma and either its corresponding anti sigma molecule, forming a biochemically inactive complex, or RNA polymerase (RNAp), forming a complex that enables transcription (figure 4A). By imposing the condition of ‘shared resources’, by which two perceptrons would compete for binding to the RNAp, the linear (ideal) decision boundary of each isolated neuron (figure 4B, in grey) exhibits nonlinearities with increasing complexity at a higher binding rate between sigma factor and the RNAp (figure 4B, in blue). A generalized model of this case study, which considers multiple competing chemical species for various resources, was proposed in [84]. A multilayer perceptron with a single hidden—promiscuous—layer was implemented and tested against a non-competing multilayer perceptron to implement Boolean logic gates (e.g. AND, XOR and XNOR) with 2 and 3 inputs. Notably, the promiscuous multilayer perceptron not only outperformed the non-competing one, but reducing the number of competing species also led to a comparable approximation of the functions to that of the non-competing multilayer perceptron. Finally, the computational capabilities of competitive dimerization networks—where monomers can bind and unbind to form either biochemically inactive (heterodimers) or active (homodimers) complexes—were addressed in [44], demonstrating that even a small network with a minimum of two interacting monomers can compute tunable, nonlinear decision boundaries.

### 2.4. Insights into the unique nature of BNN computation

It is important to highlight some key differences that emphasize the unique nature of cellular computation. Unlike ANNs, neurons across the layers of a BNN might operate at different timescales. For instance, activation and deactivation cycles, such as phosphorylation and enzymatic reactions which serve as rapid biochemical switches, operate in the order of seconds to minutes, while transcriptomic responses take from minutes to hours. This means that the systems are inherently asynchronous, with certain processes lagging behind others due to the differences in their underlying mechanisms [27]. The dynamic timescales ensure that cellular responses are highly context-dependent, as environmental stimuli and intracellular states shape the timing and the extent of these interactions. Additionally, while ANNs possess a well-defined layer-by-layer organization, the layers in BNN are not yet clearly delineated, complicating the determination of network sizes. In fact, the ‘layers’ in BNNs are not explicitly defined but rather emerge based on functional groupings, such as signalling cascades, transcriptional regulators or metabolic pathways.

## 3. Conclusions and future directions

The concept of a decision boundary at the core of cellular pattern recognition offers a promising starting point for advancing our understanding of the combinatorial logic behind signalling pathways and gene regulatory networks. The regulation of biological functions—whether involving differentiation, migration, proliferation, apoptosis or cell-type specification—is encoded in the connections and weights of the underlying BNNs, which translate combinations of inputs into these specific cellular responses. It is important to note that, although the idea of a neural network in biology was already suggested at the protein [12] and transcriptional levels [47], it was the contribution of synthetic biology that led to the convergence of concepts between machine learning and systems biology.

From an experimental perspective, the neural network framework suggests designing experiments to capture the input/output behaviour of the biological function of interest. Unlike the iterative process used to optimize synthetic gene circuits by modifying their components to achieve a desired input/output map, characterizing BNNs requires screening across a gradient of inputs and measuring the corresponding outputs. This strategy enables the empirical identification of decision boundaries. The design of experiments will then require devising strategies to continuously screen across the diversity of inputs of interest—such as by varying ligand abundances or gene expression levels—and mapping phenotypes of interest to pathway-specific genes to leverage technologies like fluorescent reporters [8] or single-cell RNA sequencing [64]. Overall, we anticipate that high-throughput methods will further enhance these characterizations [85–87].

From a theoretical perspective, several key questions remain to be addressed. For instance, what property enables a chemical reaction to compute? Could neural networks provide a broader framework for biocomputation beyond the single-cell level? This concept, initially suggested in the context of plant-specific traits [68], could be expanded using the neural network framework, as observed in bacteria at the population level [88] and during development, with functional similarities to Hopfield networks [89]. Building on the premise of BNNs as universal biological function approximators, further research should investigate how computation works in a natural biological context. This work may eventually lead to practical applications that harness the modularity of these networks for designing improved cellular therapies [37,90] and diagnostics [91].

Ultimately, studying the similarities and differences between ANNs and BNNs holds great potential for improving both fields. Insights from ANN principles can inform the design of optimization and update functions in chemical reaction networks, as well as develop synthetic mechanisms for information retention and attention in cells, ultimately leading to engineered pathways and networks for therapeutic applications. On the other hand, BNNs could inspire the development of more efficient deep learning architectures, transforming resource-intensive networks into faster, lower-power systems. In particular, biological constraints such as resource competition, growth feedback and gene expression noise—often seen as limitations to scaling synthetic gene circuits into more complex regulatory layers [92]—appear to be naturally leveraged by cells to perform more sophisticated and potentially more efficient computations with fewer nodes. Therefore, exploring the intersection of machine learning and synthetic biology can open new research avenues and drive progress in both areas.

## Data Availability

Python scripts (Jupyter notebook format) to reproduce the simulations displayed in the figures are available in our Github repository [93].
